# The Conquest of Disease

**Published:** 1923-03

**Authors:** 


THE
### HOSPITAL and HEALTH
REVIEW

### THE CONQUEST OF DISEASE.

IT is one of the defects of statistics that they are commonly stale. But nothing is really out-of-date until it is superseded by something newer, and although the Registrar-General's Annual Statistical Review, which has just been issued, relates to 1921, the figures are, in their way, as instructive as if they came down to the end of last December. Nevertheless it would be useful if this conspectus of the vital statistics of the country could be produced with a delay of rather less than thirteen months or so. Now that the figures are available there is every reason to be thankful for them. They not only indicate a remarkable and progressive improvement in the public health, but establish the splendid certainty that the "crude" death-rate—12·1 per thousand—is the lowest ever recorded since civil registration was instituted on the very morrow of Queen Victoria's accession. The outstanding fact is that the death-rate in 1921 was not much more than half the average of the decade 1841-1850. Not the least interesting detail of these really romantic figures is that whereas between the decade ending in 1850 and that ending in 1880 the death-rate fell by only one per thousand, in the first decade of the present century it fell by exactly six per thousand. Had it remained in 1921 what it was in 1841-50 there would have been nearly 850,000 deaths in that year instead of 458,629.

This diminution, growing progressively more rapid as we reach the present time, is no mere accident. It is the result, not of legislation, though that has played an important part in the improvement, but of the laborious and self-sacrificing work of doctors and nurses, sanitary officers and the vast number of laymen and women who have helped to "tighten up" the local health administration of the country. Research in the laboratory, too, has produced brilliant results in the war upon disease, and it is impossible even to turn over the pages of the Registrar General's voluminous review without realising that there is no department of life in which quicker and more solid progress has been made of late years than in that which deals with the public health. We have said that the "crude" death-rate for 1921 was 12·1 per thousand, but the "standardised" rate was only 11·5, which again is the lowest ever recorded in this country. Never before have the rates been so low at all age-groups up to 65. The most remarkable, and in many ways the most gratifying, fall was in infant mortality, which was the lowest yet recorded save in 1920. At 83 per thousand it was 25 per thousand lower than the average rate of the five pre-war years. The rate was, however, extraordinarily variable. While it was only 54 in Stoke Newington, it was 111 in Shoreditch ; at Stoke-on-Trent it reached the terrible total of 135. It would seem that this great combination of Pottery towns is in urgent need of a Mission of Help to Mothers.

Let us not, however, rejoice too soon. We already know that the provisional death-rate for 1922 in England and Wales was a fraction higher than in 1921, and that in London, thanks to influenza, it was nearly one per thousand above that of the country at large. We have carried the war against disease a long way into the enemy's country ; but there are still vast conquests to be made. Heart disease, cancer, tuberculosis, pneumonia, and bronchitis, together, still account for nearly one half of the annual deaths. Heart disease is more deadly even than cancer, though less generally dreaded ; pneumonia is only one per cent. less destructive than tuberculosis ; bronchitis is nearly as fatal. Nor should we forget than so recently as 1918 influenza killed far more people than cancer and tuberculosis together. Happily there has been a rapid fall in the mortality from this scourge from the peak of 1918, until in 1921 there were barely 9,000 deaths from this cause to deplore in place of the terrible total of more than 112,000 in the worst year. We have not yet learned the secret of these epidemics, which have been more or less seriously recurrent for more than a century. But if influenza is an occasional, though deadly, enemy, the other diseases we have enumerated are constant in the heavy toll they take of human life. No one of them will be conquered suddenly, though it is beyond doubt that we shall in time know enough of their causation to be able to deal with them with relative success. Meanwhile it is the business, not merely of medical men and specialists in sanitation, but of all of us, to see to it that the conditions of life and the surroundings of employment are so substantially ameliorated that the more fatal forms of disease find a less favourable seed-bed, and that it becomes easier to die of " old age " than from causes which it is in the power of science to mitigate and control.

